# Quantifying and Optimizing Single-Molecule Switching Nanoscopy at High Speeds

**DOI:** 10.1371/journal.pone.0128135

**Published:** 2015-05-26

**Authors:** Yu Lin, Jane J. Long, Fang Huang, Whitney C. Duim, Stefanie Kirschbaum, Yongdeng Zhang, Lena K. Schroeder, Aleksander A. Rebane, Mary Grace M. Velasco, Alejandro Virrueta, Daniel W. Moonan, Junyi Jiao, Sandy Y. Hernandez, Yongli Zhang, Joerg Bewersdorf

**Affiliations:** 1 Department of Cell Biology, Yale University School of Medicine, New Haven, Connecticut, United States of America; 2 Department of Biomedical Engineering, Yale University, New Haven, Connecticut, United States of America; 3 Integrated Graduate Program in Physical and Engineering Biology, Yale University, New Haven, Connecticut, United States of America; 4 Yale College, Yale University, New Haven, Connecticut, United States of America; 5 Institute for Molecular Biophysics, The Jackson Laboratory, Bar Harbor, Maine, United States of America; 6 Department of Physics, Yale University, New Haven, Connecticut, United States of America; 7 Department of Mechanical Engineering and Material Science, Yale University, New Haven, Connecticut, United States of America; 8 Department of Molecular Biophysics and Biochemistry, Yale University, New Haven, Connecticut, United States of America; University of California, Berkeley, UNITED STATES

## Abstract

Single-molecule switching nanoscopy overcomes the diffraction limit of light by stochastically switching single fluorescent molecules on and off, and then localizing their positions individually. Recent advances in this technique have greatly accelerated the data acquisition speed and improved the temporal resolution of super-resolution imaging. However, it has not been quantified whether this speed increase comes at the cost of compromised image quality. The spatial and temporal resolution depends on many factors, among which laser intensity and camera speed are the two most critical parameters. Here we quantitatively compare the image quality achieved when imaging Alexa Fluor 647-immunolabeled microtubules over an extended range of laser intensities and camera speeds using three criteria – localization precision, density of localized molecules, and resolution of reconstructed images based on Fourier Ring Correlation. We found that, with optimized parameters, single-molecule switching nanoscopy at high speeds can achieve the same image quality as imaging at conventional speeds in a 5–25 times shorter time period. Furthermore, we measured the photoswitching kinetics of Alexa Fluor 647 from single-molecule experiments, and, based on this kinetic data, we developed algorithms to simulate single-molecule switching nanoscopy images. We used this software tool to demonstrate how laser intensity and camera speed affect the density of active fluorophores and influence the achievable resolution. Our study provides guidelines for choosing appropriate laser intensities for imaging Alexa Fluor 647 at different speeds and a quantification protocol for future evaluations of other probes and imaging parameters.

## Introduction

The family of single-molecule switching nanoscopy techniques, such as photoactivated localization microscopy (PALM) [1], fluorescence photoactivation localization microscopy (FPALM) [2], stochastic optical reconstruction microscopy (STORM) [3], ground state depletion microscopy followed by individual molecule return (GSDIM) [4] and direct STORM (dSTORM) [5], achieves sub-diffraction limit resolution based on stochastic switching of photoswitchable probes and precise localization of individual molecules. For simplicity, we will refer to this group of techniques as SMSN (short for single-molecule switching nanoscopy). Typically, to obtain a SMSN image with ~20–40 nm resolution, one needs to acquire 10,000 to 100,000 camera frames to record the positions of millions of molecules. Using the full 512 x 512 pixels of the commonly used EMCCD cameras, the camera speed in SMSN is limited to ~100 frames per second (fps). The data acquisition process therefore can take from hundreds of seconds to tens of minutes. Higher camera speeds are possible at the expense of smaller fields of view [6–8]. Recently, the use of scientific complementary metal-oxide semiconductor (sCMOS) camera technology in SMSN has enabled imaging at high speeds even over large fields of view [9] and camera speeds up to 3,200 fps have been demonstrated in biological SMSN applications [10].

**Fig 1:**
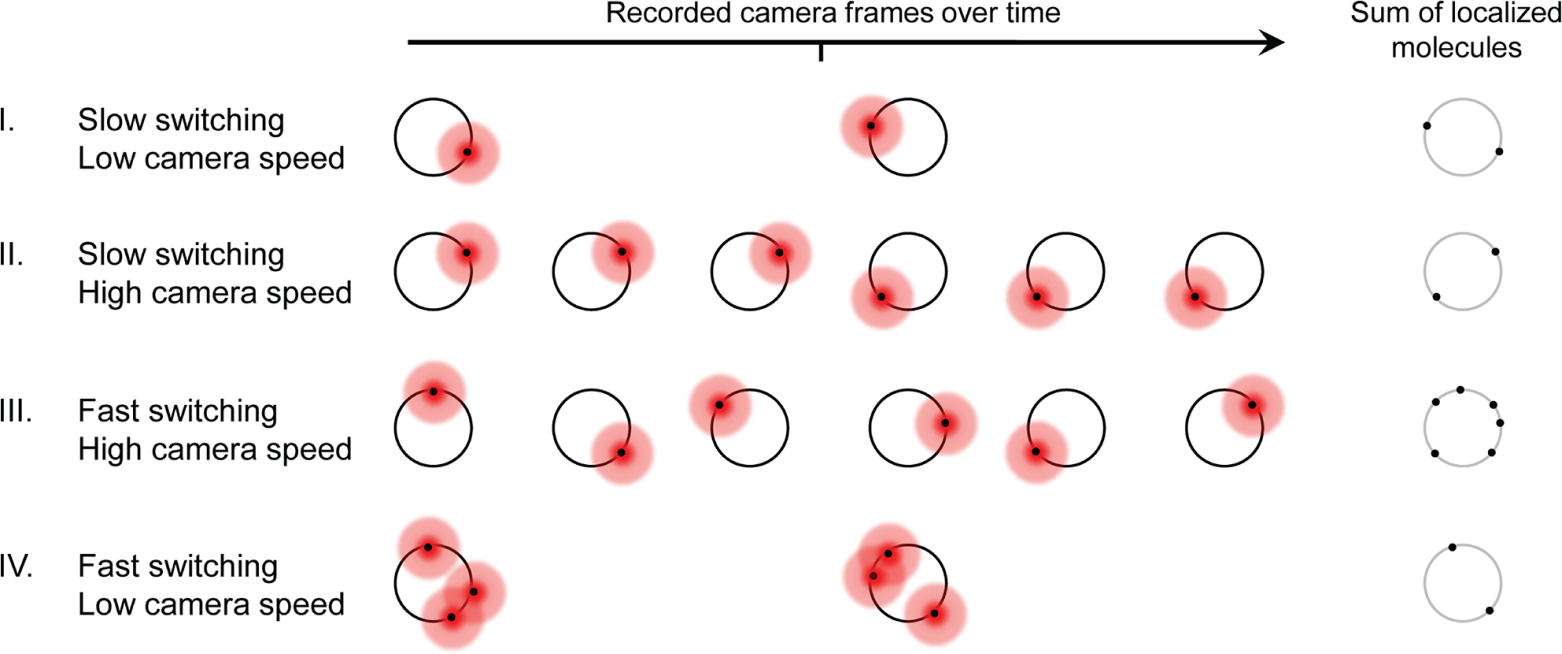
The influence of single-molecule switching kinetics and camera frame rate on SMSN imaging. A schematic shows the accumulation of localized molecules over time for different scenarios. The fluorescence emission (red circles) of each molecule labeling a ring-like structure is fit to yield its position (black dots). Both fast switching kinetics and high camera speed are required for the most efficient localization of molecules (case III).

Previous studies have shown that the photoswitching kinetics of fluorescent probes, such as Cy5 and Alexa Flour 647, can be accelerated with higher laser intensities [5, 11]. High laser intensities, in combination with fast camera speeds, haven been applied to significantly shorten the data acquisition time of SMSN [7, 8, 10, 12] (**Fig 1**). On the other hand, these changes also influence, for example, the number of photons detected per molecule and the background, both of which strongly affect localization precision and thus the image quality. Laser intensity and camera speed are therefore two key parameters affecting the spatial and temporal resolution of SMSN.

Various combinations of laser intensities and camera speeds have been used in SMSN imaging at both high speeds and conventional speeds [7, 8, 10, 12–15]. However, it is difficult to directly compare these studies in an unbiased manner because diverse samples, varied data processing routines and different criteria for image quality assessment were used. Therefore, the selection of optimal imaging conditions has been a challenge since a comprehensive study that systematically compares the achievable SMSN image quality over a large range of imaging parameters has not been available.

To address this problem, we performed a quantitative study that compares different metrics of image quality over two orders of magnitude of laser intensities and camera speeds in both short and long term imaging (**Table 1**). We focused on imaging Alexa Fluor 647 in an imaging buffer containing the thiol compound β-mercaptoethanol and an enzymatic oxygen-scavenging system because it is widely used in SMSN[14]. We designed our experiments to answer the following questions:

1. Does imaging at high speeds reduce localization precision?
2. What are the imaging parameters that achieve optimal spatial and temporal resolution?
3. Does increased laser intensity compromise the maximum achievable density of localized molecules?

**Table 1:** Overview of experimental design.

Sample	Imaging parameters		Quantitative criteria
Alexa Fluor 647-immunolabeled microtubules in a thiol-based buffer	642-nm laser intensities: 1.0, 1.9, 3.9, 7.8, 16, 31, 62, 97 kW/cm^2^	Camera speeds: 50, 100, 200, 400, 800, 1600 fps	Localization precision
			Localization density
			Image resolution

## Results

### Experimental design

We prepared Alexa Fluor 647-immunolabled microtubules embedded in an imaging buffer containing β-mercaptoethanol, glucose oxidase and catalase as standard samples (see **Methods** for details). We imaged these samples with 48 pairs of imaging parameters chosen from eight laser intensities (1.0, 1.9, 3.9, 7.8, 16, 31, 62, and 97 kW/cm^2^) of 642-nm light and six camera speeds (50, 100, 200, 400, 800, and 1,600 fps). To minimize the influence of sample variation, arising potentially from cell-to-cell variability or differences in aging of the imaging buffer, we acquired four data sets for each condition on four different days with different samples. On each day, we recorded 48 data sets corresponding to the 48 conditions in randomized order. For consistency and easier interpretation of the data, we did not use additional activation laser light in the above experiments.

To quantify the image quality achieved at different conditions, we extracted the localization precision, density of localized molecules and image resolution based on Fourier Ring Correlation (FRC) [16] from each data set (see **Methods** for details). Briefly, we first generated an initial list of single-emitter localization events *E*_*init*_ from the raw camera frames using previously developed algorithms [10]. Next, we identified localization events from this list that occurred in close spatial and temporal proximity (see **Methods** for details). Since the probability that neighboring molecules are activated within the same short time period is negligibly low, we considered these events to stem from the same molecule that stayed on for multiple frames and/or blinked rapidly. For these events, we extracted the corresponding pixel values from the raw camera frames, added them together and localized again with an adapted noise model (see **Methods** for details). Finally, after rejecting localization events with localization uncertainties larger than 25 nm, we generated a new list of localization events *E*_*eff*_, which was used to calculate the values of the average localization precision and density reported below and to reconstruct SMSN images.

### The dependence of localization precision and density on speed and intensity

**Fig 2:**
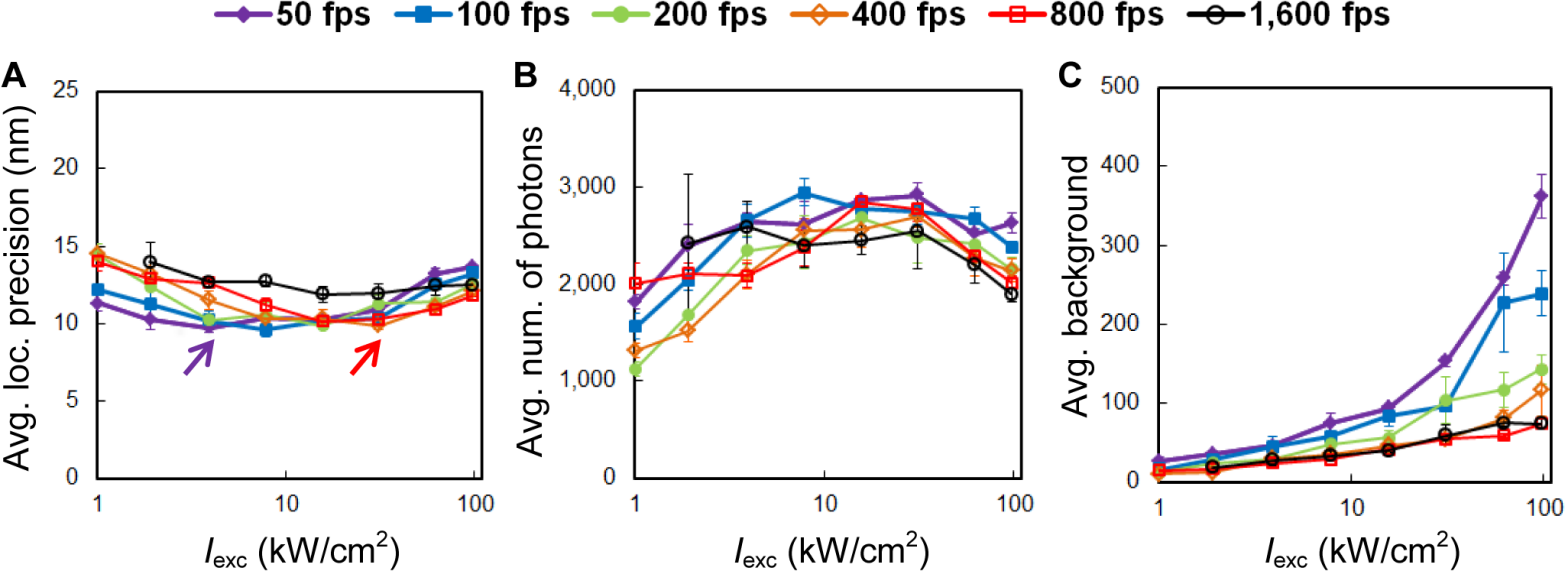
Localization precision, number of detected photons and background. (**A**) The average localization precision as a function of 642-nm laser intensity measured at different camera frame rates. Optimal localization precision of ~10 nm can be achieved at both conventional speed (50 fps, purple arrow) and high speed (800 fps, red arrow). (**B, C**) The average number of detected photons per localization event *E*_*eff*_ (**B**) and the average number of background photons per localization event *E*_*eff*_ per pixel (**C**) at corresponding conditions.

Localization precision is one of the determinants of the achievable image resolution in SMSN [17]. We found that average localization precision, calculated based on the Cramér-Rao Lower Bound, has only a weak dependence on speed and intensity (**Fig 2A**). For the tested camera speeds between 50 and 800 fps, the best localization precisions achieved were similar (~10 nm) when using optimized laser intensities. The faster the camera speed, the higher the intensity is required to achieve optimal localization precision. For 1,600 fps, the best localization precision achieved was slightly worse (~12 nm) than for lower frame rates (~10 nm). Furthermore, since localization precision is largely dependent on photon number and background noise [18], we analyzed the average number of detected photons per localization event *E*_*eff*_ (**Fig 2B**) and the average background per localization event *E*_*eff*_ (**Fig 2C**) at different conditions. We found that, at low intensities (1.0–3.9 kW/cm^2^), low acquisition speeds at 50–200 fps provided higher photon numbers and thus better localization precisions than high speeds (400–1,600 fps). On the other hand, with high intensities (31–97 kW/cm^2^), low speeds led to accumulation of higher background which resulted in worse localization precisions.

**Fig 3:**
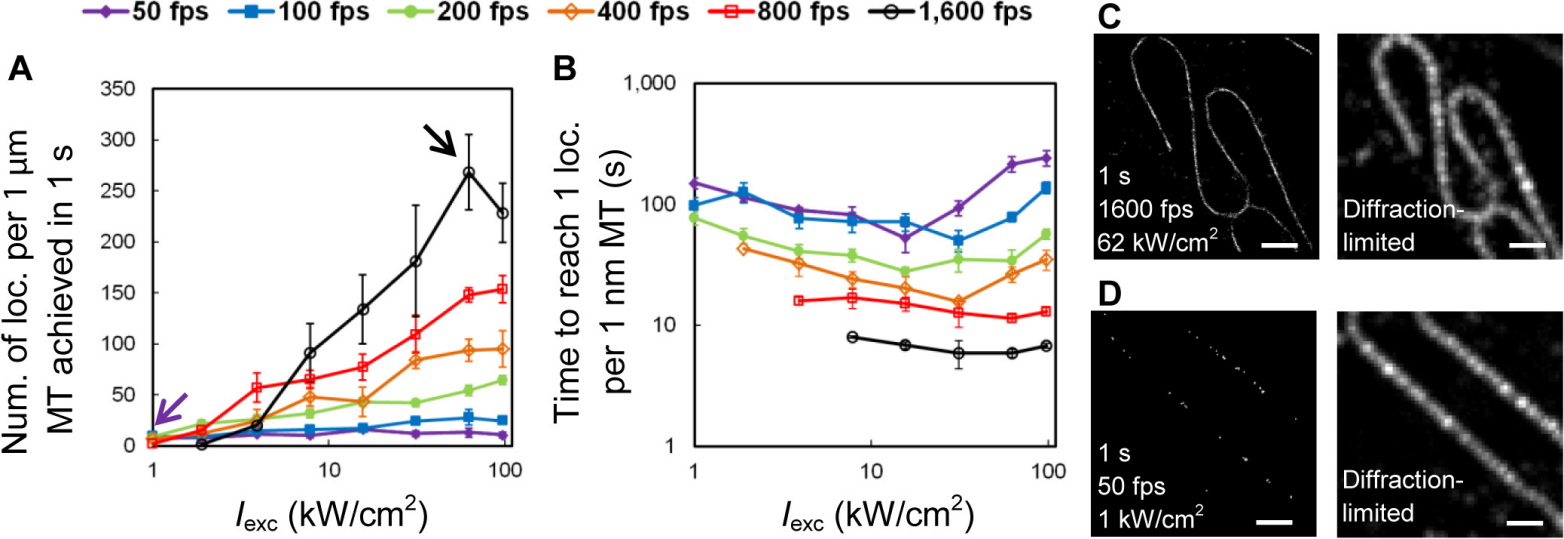
Density of localized molecules. (**A**) Localization density, described as the average number of localization events per 1 μm length of microtubule (MT), obtained in the first 1 s of data acquisition. High-speed SMSN (black arrow) achieves 35 times higher localization density than SMSN at conventional speed (purple arrow). (**B**) Data acquisition time required to reach the target density of 1 localization event per 1 nm length of MT. (**C, D**) Example SMSN images of Alexa Fluor 647-immunolabeled microtubules reconstructed from data acquired in the first 1 s at both high-speed (**C**) and conventional speed (**D**) conditions. Scale bars: 1 μm.

The density of localized molecules is another factor that affects the resolution in SMSN since structures can only be resolved if the average distance between localized molecules lies significantly below the structure size. First, we compared the average density of localization events *E*_*eff*_ achieved within the first 1 s of data acquisition at all tested conditions (**Fig 3A**). We found that the combination of high intensities (7.8–97 kW/cm^2^) and high speeds (800–1,600 fps) can boost the localization density by 5–25 times and achieve super-resolution imaging within 1 second. **Fig 3**shows two example images reconstructed from data acquired in 1 second at 1,600 fps and 62 kW/cm^2^ (**Fig 3C**), and at 50 fps and 1 kW/cm^2^ (**Fig 3D**) respectively. While the former image can already resolve the microtubule structure in detail, the latter image shows only scattered molecule positions. For a more quantitative comparison, we introduce the temporal resolution measure *t*_*ρ* = 1/nm_ as the time required to reach a target density of 1 localization event per nm length of microtubule. We found that at high-frame rate, high-intensity conditions (1,600 fps and 62 kW/cm^2^), *t*_*ρ* = 1/nm_ is 32-fold shorter than for the conventional imaging conditions (50 fps and 1 kW/cm^2^): *t*_*ρ* = 1/nm_ = 5 s vs. *t*_*ρ* = 1/nm_ = 160 s (**Fig 3B**).

**Fig 4:**
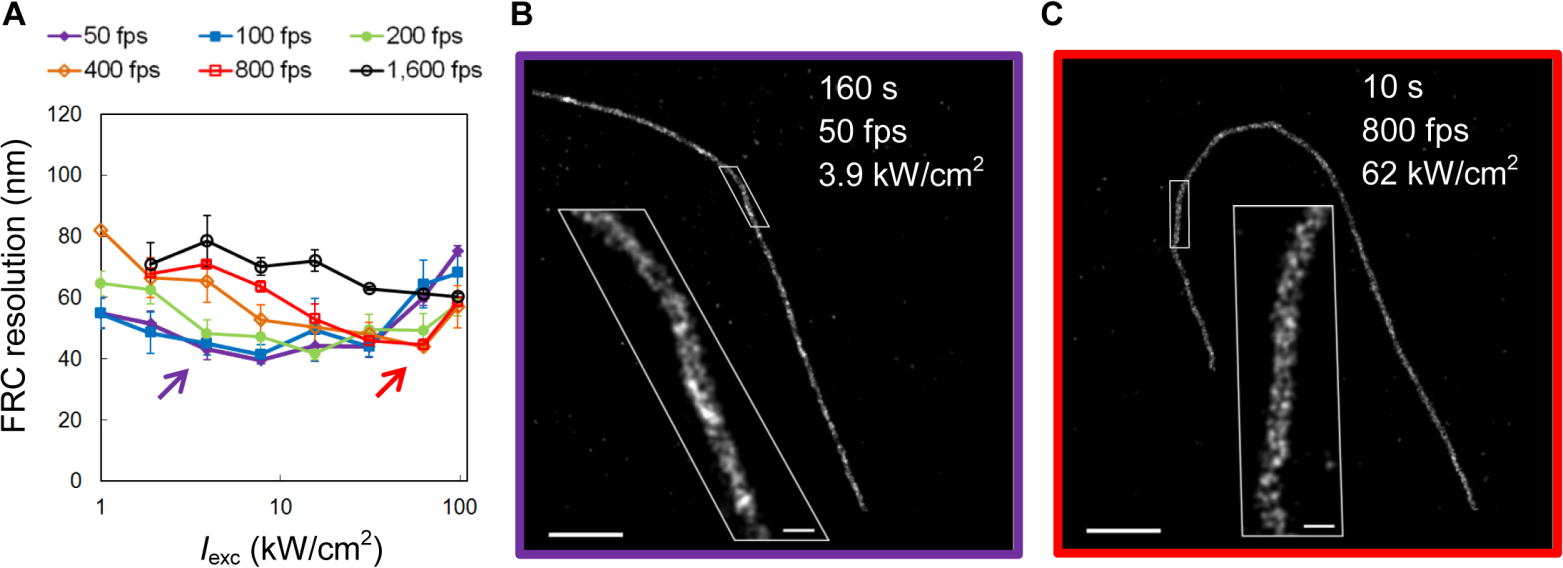
Image resolution comparison of high-speed and conventional SMSN. (**A**) FRC resolution values calculated from SMSN images reconstructed from 20,000 raw camera frames acquired at different camera speeds and laser intensities. (**B**, **C**) Example SMSN images of Alexa Fluor 647-immunolabeled microtubules from conventional (purple arrow, **B**) and high-speed SMSN (red arrow, **C**) demonstrate comparable image quality with ~40 nm FRC resolution. Insets show further magnification of the white box areas respectively. The images are reconstructed from data acquired in 160 s at 50 fps and 3.9 kW/cm^2^ with a density of 1,572 localizations per 1 μm MT (**B**), and data acquired in 10 s at 800 fps and 62 kW/cm^2^ with a density of 1,091 localizations per 1 μm MT (**C**). Scale bars: 1 μm; inset 100 nm.

We next assessed the image quality achieved at different conditions using the FRC method, which combines localization precision and density in a single measure. The FRC resolution values of images reconstructed from 20,000 frames each show that imaging at high-frame rate, high-intensity conditions (800 fps and 31–62 kW/cm^2^) achieves a FRC resolution (~40 nm) that is comparable to that attained at conventional imaging conditions (50 fps and 3.9 kW/cm^2^) (**Fig 4A**). Consequently, reconstructed images from both conditions exhibit similar detail and quality for the same number of camera frames (**Fig 4B and 4C**). However, data recording time in the high-speed case was shortened by a factor of 16 (**S1 and S2 Movies**).

### The effect of photobleaching on localization density

In the previous section, we showed that imaging at 800–1,600 fps with 16–97 kW/cm^2^ of 642-nm light improves the temporal resolution by 16–32 times without compromising image quality. However, high intensities also raise concerns about increased photobleaching. We therefore looked into how photobleaching affects the achievable localization density over longer time courses. We imaged our microtubule samples with three pairs of imaging parameters (3.9 kW/cm^2^ and 50 fps, 31 kW/cm^2^ and 800 fps, and 62 kW/cm^2^ and 800 fps) for 160,000–400,000 frames, both with and without 405-nm activation light. The 405-nm light is used to return Alexa Fluor 647 molecules from the reversible dark states back to the emissive state.

**Fig 5:**
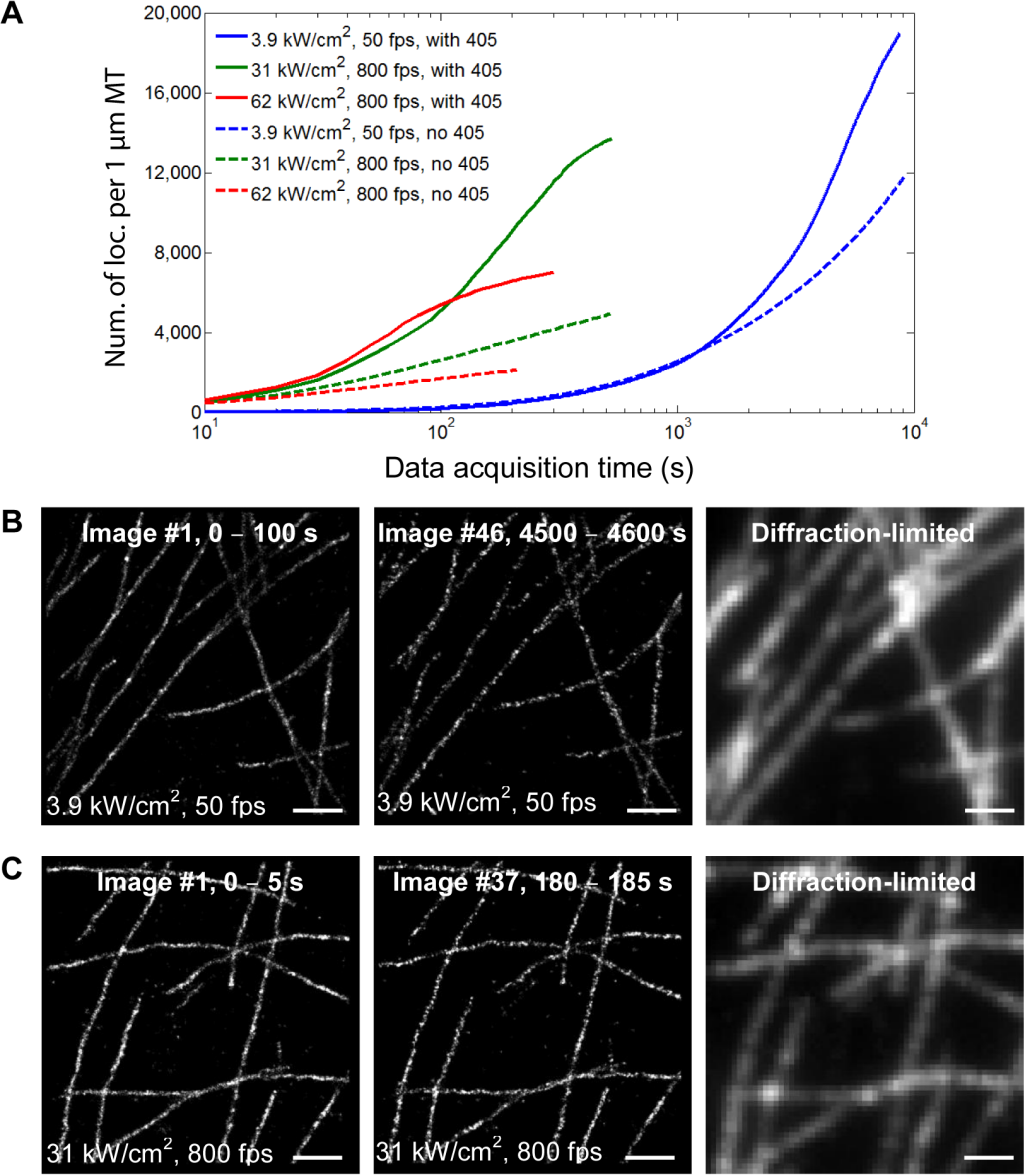
Effect of photobleaching on localization density at different laser intensities. (**A**) Localization density accumulated with three pairs of imaging parameters over long term imaging (160,000–400,000 frames per data set). (**B, C**) Total number of SMSN images obtained before the spatiotemporal resolution decreases due to photobleaching. (**B**) When imaging at 3.9 kW/cm^2^ and 50 fps with 405-nm light, 46 SMSN images were generated with a localization density of ~800 localizations per 1 μm MT and temporal resolution of 100 s per image. (**C**) When imaging at 31 kW/cm^2^ and 800 fps with 405-nm light, 37 SMSN images were generated with the same localization density and improved temporal resolution of 5 s per image. Scale bars: 1 μm.

We found that by adding 405-nm light, irrespective of the 642-nm laser intensity, the final achieved localization density is consistently increased two to threefold when imaging for long times (**Fig 5A**). Independent of the 405-nm light, higher intensities of the 642-nm light led to lower total numbers of localization events, which suggests the existence of a non-linear photobleaching component. However, this effect is not dominant given that the 16-fold increase of the 642-nm light intensity from 3.9 to 62 kW/cm^2^ led only to a ~3-fold reduction in the maximum achieved density from 1.9 x 10^4^ to 0.7 x 10^4^ localizations per 1 μm length of microtubule. For practical considerations, we also compared the number of high-quality SMSN images that can be generated before the sample is severely bleached. When imaging at 3.9 kW/cm^2^ and 50 fps with 405-nm light, we were able to generate a SMSN image every 100 s with the target density of 800 localizations per 1 μm length of microtubule. In total, 46 SMSN images were generated before the spatiotemporal resolution decreased due to photobleaching (**Fig 5B**). For imaging at 31 kW/cm^2^ and 800 fps, we could generate SMSN images with the same localization density every 5 s and obtained 37 images in total (**Fig 5C**). Our results show that, with 405-nm activation light, imaging at high excitation intensities such as 31 kW/cm^2^ does not severely affect the final achievable localization density while still increasing the temporal resolution by more than one order of magnitude.

### Simulation of SMSN imaging at different speeds and intensities

The density of active fluorophores, here defined as molecules in the fluorescence-emitting ON-state, is critical for the spatial and temporal resolution in SMSN. Too high of a density will cause the images of individual molecules to overlap and affect the localization precision as well as the rejection rate of localization events. On the other hand, if the density is too low, longer times are required to reach the target localization density.

To understand how photoswitching properties at different intensities affect the density of active fluorophores, we carried out single-molecule experiments to determine the switching kinetics of Alexa Fluor 647 and developed a simulation tool that can create artificial SMSN raw data based on the measured kinetics.

**Fig 6:**
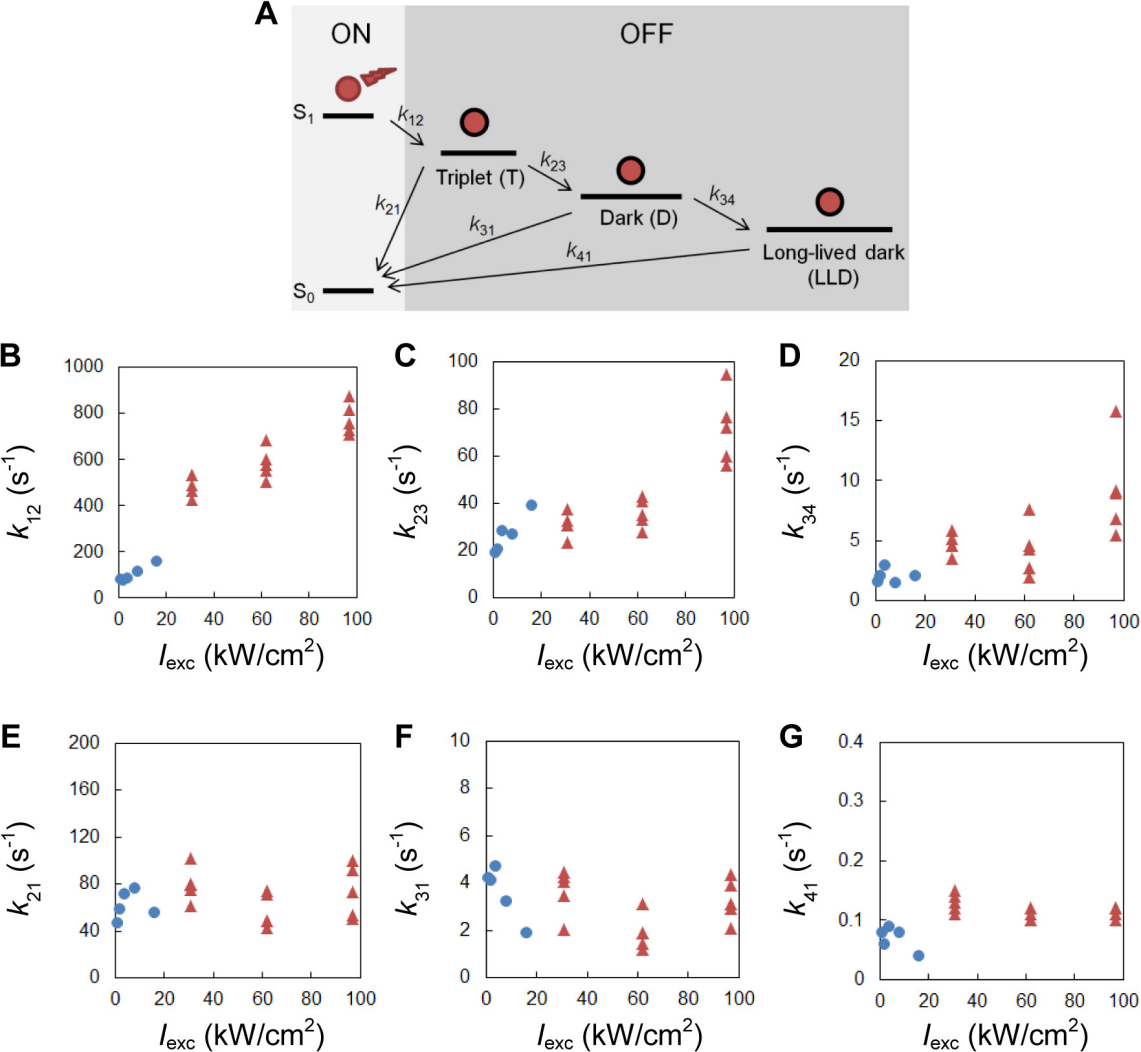
Effect of laser intensity on Alexa Fluor 647 photoswitching kinetics. (**A**) Model of Alexa Fluor 647 photoswitching mechanism [20, 21]. Upon irradiation, the fluorophore can undergo intersystem crossing and switch from the fluorescence-emitting ON-state to the triplet state with rate *k*_*12*_. The triplet state (T) can either recover to the singlet ground state or react with a thiolate to form the radical anion of the fluorophore (dark state, D). The dark state can be oxidized to recover to the singlet ground state or form a thiol adduct [22] (long-lived dark state, LLD). (**B—G**) Photoswitching rates at different excitation laser intensities extracted from single-molecule experiments. At low intensities (1.0–16 kW/cm^2^, blue dots), data were recorded at 200 fps to allow for high temporal resolution and high signal-to-noise ratio. At high intensities (31–97 kW/cm^2^, red triangles), data were recorded at 800 fps because the ON-state lifetime is reduced to a few milliseconds and requires higher temporal resolution.

Previous studies suggested that the local environment of the fluorophore such as the oxygen level in the buffer and the interaction between the fluorophore and the labeled molecule could affect its photoswitching properties [19]. To determine the switching kinetics in our sample, we imaged single Alexa Fluor 647-labeled antibodies that were immobilized on coverslips, at eight different intensities ranging from 1.0 to 97 kW/cm^2^ (see **Methods** for details). For each intensity, we recorded ~1,600–6,000 events from ~ 300–1,400 molecules where a molecule switched reversibly from a fluorescent ON-state to a non-fluorescent OFF-state. We found that the distributions of ON-times (i.e. the dwell times in the ON-state) from all the molecules were fit well by an exponential decay function. On the other hand, the OFF-times (i.e. the dwell times in the OFF-states) were spread out over a wide range from several milliseconds to tens of seconds. The distributions of OFF-times could not be fit by a single exponential function, but rather a sum of at least three exponential functions. Examples of an OFF-time distribution with fit and OFF-time distributions at different intensities are shown in **Figure A and Figure B in S1 File,** respectively. Our observation is consistent with the photoswitching model (**Fig 6A**) proposed by Vogelsang *et al*. and van de Linde *et al*. [20, 21]. Based on this model, we further derived the distributions of ON/OFF-times as functions of the photoswitching rates (see **Methods** for details). From the derived functions and the ON/OFF-times measured in the single-molecule experiments, we calculated the on and off-switching rates (defined as shown in **Fig 6A**) at different intensities. We found the off-switching rate to strongly increase with excitation laser intensity (**Fig 6B**), and the on-switching rates to be nearly independent of excitation laser intensity (**Fig 6E–6G**) over the intensity range tested.

**Fig 7:**
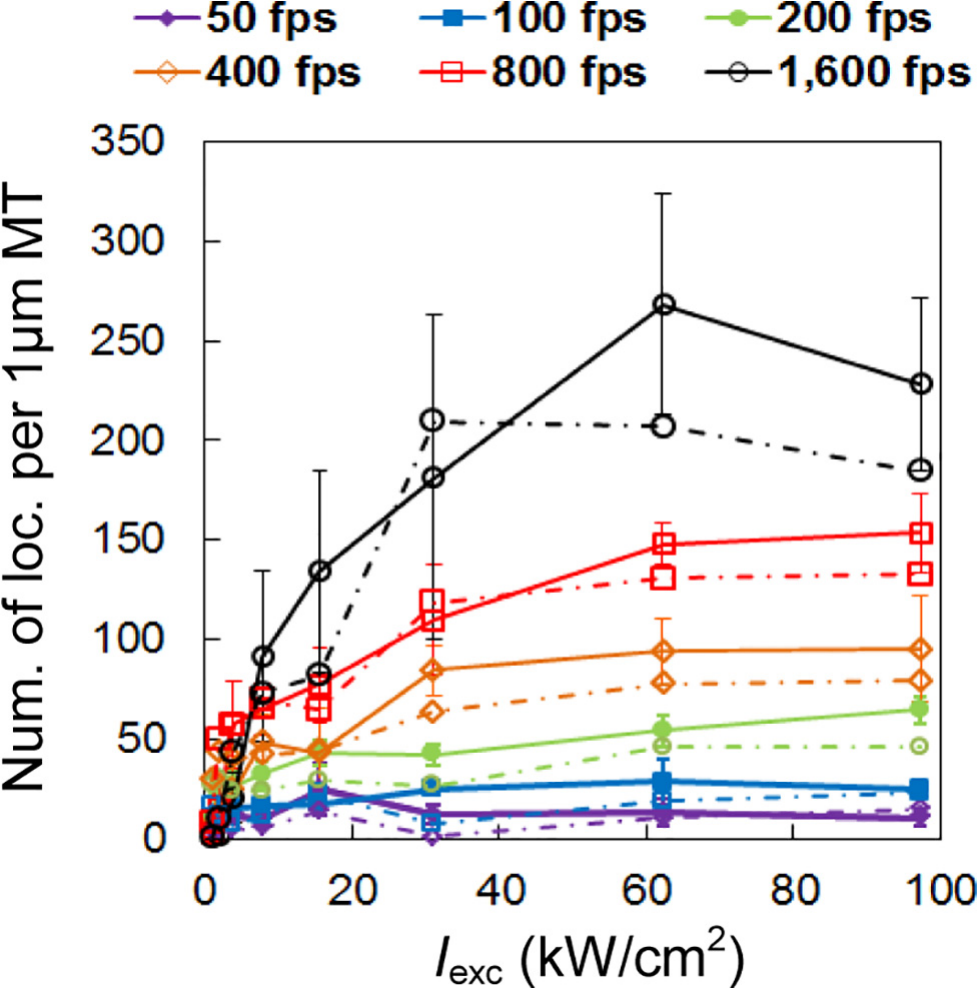
Simulation reproduces experimentally-observed dependence of localization density on laser intensity and frame rate. Localization density obtained in the first 1 s of data acquisition from both experiment (solid lines; extracted from the same raw data as **Fig 3A**) and simulation (dash-dot lines) data. Alexa Fluor 647 photoswitching model and switching rates used in the simulation were reported in **Fig 6**. The structure of artificial microtubules used in our simulations is illustrated in **Fig 8A**.

Using the photoswitching rates obtained above, we developed algorithms (**S1 Software**) to simulate SMSN imaging of artificial microtubules at the same imaging conditions as used in the quantification study (see **Methods** for details). We processed the simulated images the same way as the experimental data and calculated the density of localized molecules achieved in the first 1 s. The simulation results we obtained correspond well with our experimental data (**Fig 7**).

**Fig 8:**
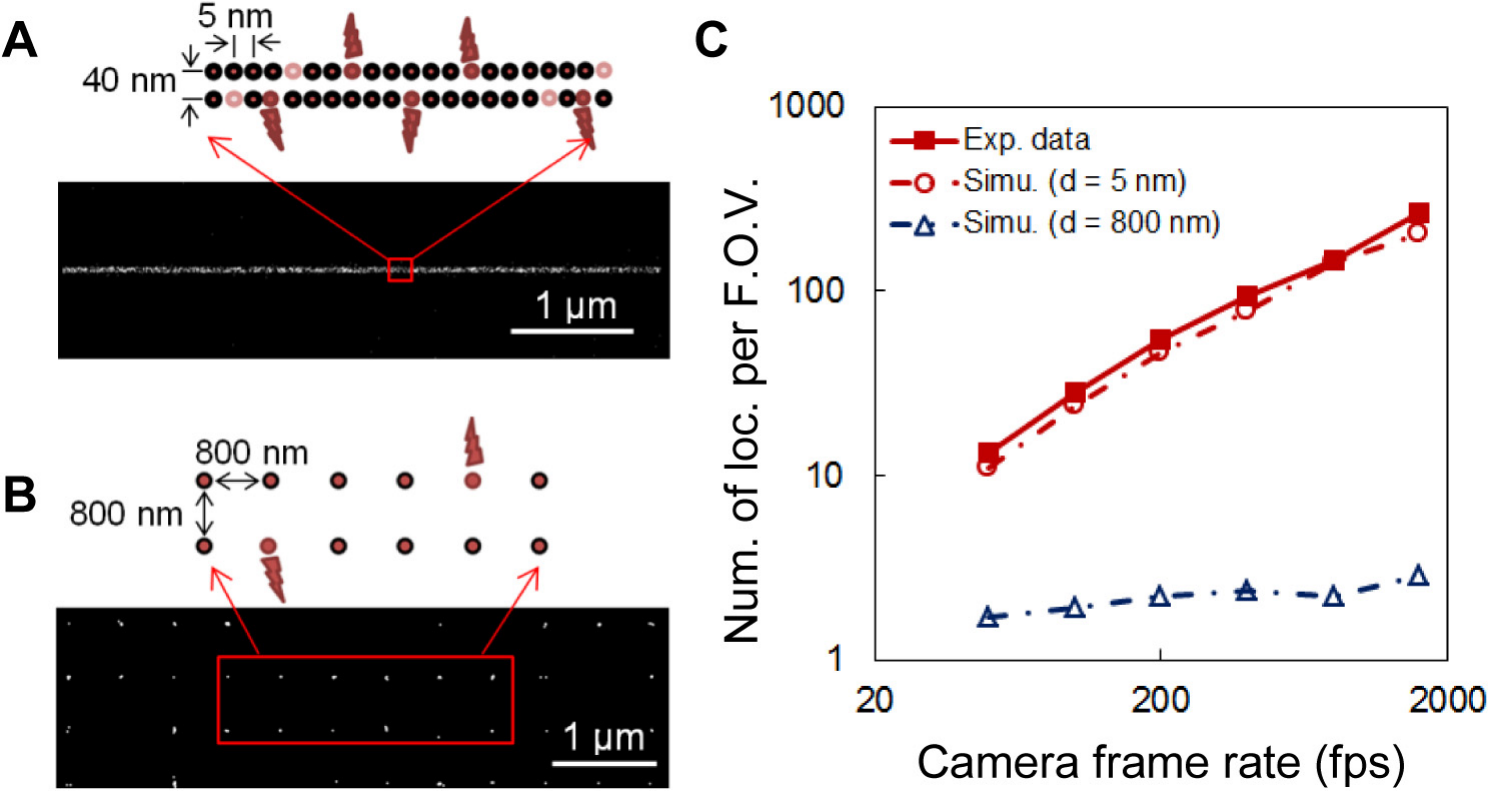
Effect of camera speed on localization density for samples with different labeling densities. Schematic diagram and simulated SMSN images of an artificial microtubule (**A**) and an artificial grid structure with sparse labeling (**B**). (**C**) Number of localizations obtained per field of view (F.O.V.) within the first 1 s of data acquisition from both experimental data (extracted from the same raw data as **Fig 3 A**, *I*_*exc*_ = 62 kW/cm^2^,) and simulated SMSN imaging of an artificial microtubule with dense labeling (as illustrated in **A**) as well as a grid structure with sparse labeling (as illustrated in **B**).

In both the experiment and the simulation results (**Fig 7**), we observed a strong correlation between the localization density and the camera speed. We reasoned that this correlation was not caused by repeated localizations of the same molecule that stayed on for multiple frames because, as described earlier, such localizations had already been identified and combined in data analysis. To investigate the cause of this correlation, we looked into the raw images recorded at different camera speeds. We found that, at low speeds, there is a higher probability that multiple fluorophores from within a diffraction-limited area are activated during the camera exposure time, which leads to the overlap of their images. These overlapping signals are then rejected by the localization algorithm, which results in low localization densities (**Figure C in S1 File**). To test this hypothesis, we simulated SMSN imaging of artificial structures with high and low labeling densities (**Fig 8A and 8B**) at different camera speeds. We expected that in the case of low labeling density, signals from neighboring molecules are much less likely to overlap and that imaging at low speeds should achieve similar localization densities as at high speeds. This was confirmed by the simulation results (**Fig 8C**).

**Fig 9:**
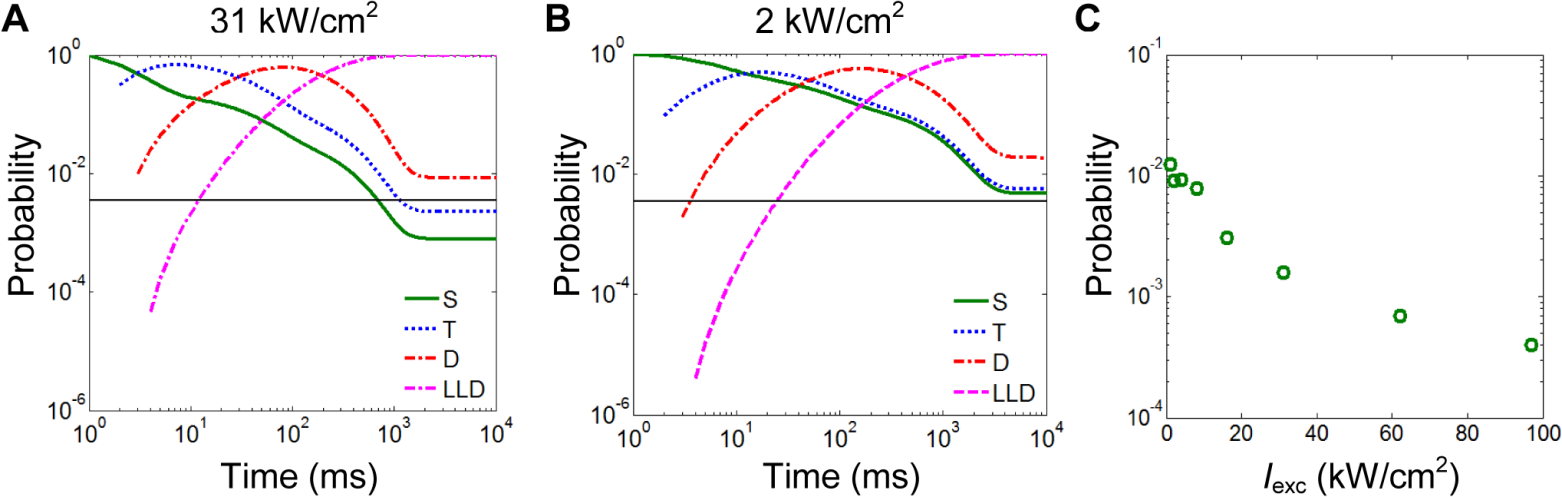
Effect of photoswitching dynamics on the density of active fluorophores. (**A, B**) Fraction of Alexa Fluor 647 molecules in the ON-state (the singlet state, S) and the OFF-states (the triplet state, T, the dark state, D and the long-lived dark state, LLD) over time upon irradiation with 31 kW/cm^2^ (**A**) and 2 kW/cm^2^ (**B**) of 642-nm light. The horizontal black lines mark the threshold for the optimal fraction of fluorophores in the ON-state, which corresponds to the optimal density of active fluorophores (1 active fluorophore per 700 nm length of microtubule) in the case of an artificial microtubule as illustrated in **Fig 8 A**. (**C**) The fraction of fluorophores in the ON-state at equilibrium at different excitation intensities.

Finally, we investigated how the photoswitching dynamics at different laser intensities affect the density of active fluorophores over the first seconds of imaging. With a given labeling density, the density of active fluorophores is determined by the fraction of fluorophores in the ON-state. Based on the photoswitching model and kinetics described in **Fig 6**, we calculated the distribution of fluorophores in the four photophysical states over time upon irradiation with 642-nm light (**Fig 9**). Initially, the majority of fluorophores are in the ON-state, which leads to a high density of active fluorophores and makes identifying individual molecules difficult. At high laser intensities (31 kW/cm^2^, **Fig 9A**), the fraction of fluorophores in the ON-state quickly drops below the threshold (marked as the horizontal black lines in **Fig 9A and 9B**), where the density of active fluorophores is optimal so that individual molecules can be identified and localized effectively. At low intensities (2 kW/cm^2^, **Fig 9B**), the fraction of fluorophores in the ON-state not only decreases much more slowly, but its equilibrium value is still above the threshold which leads to inefficient single-molecule localization. In practice, this equilibrium value can be further reduced by photobleaching to improve the localization efficiency. However, since the effect of photobleaching is not included in our calculation, the transition of fluorophores between ON/OFF-states reaches equilibrium after ~ 1–2 s. Furthermore, our result shows that, at equilibrium, the fraction of fluorophores in the ON-state decreases with increasing excitation laser intensity (**Fig 9C**).

## Discussion

In summary, we quantified the image quality achieved in SMSN imaging at camera frame rates ranging from a conventional speed of 50 fps to 32-times its value (1,600 fps) and for laser intensities ranging over two orders of magnitude (10^0^–10^2^ kW/cm^2^). We found that a combination of high laser intensities (16–97 kW/cm^2^) and high frame rates (800 fps) achieves uncompromised image resolution in time periods 5–25 folds shorter than the conventional SMSN imaging conditions (1 kW/cm^2^, 50 fps). Furthermore, we studied the dependence of the photoswitching kinetics of Alexa Fluor 647 on the excitation intensity with single-molecule experiments. Based on the switching kinetics obtained experimentally, we could simulate the SMSN imaging process and specifically test how the density of active fluorophores affects the achievable SMSN image quality for our range of experimental conditions.

Our finding that SMSN imaging can be accelerated without compromising the image quality is especially important for high-throughput SMSN imaging: faster imaging speeds allow recording of much larger numbers of samples than previously possible. This, in turn, enables more complex experiments that require large numbers of cells to be imaged at multiple conditions.

In this work, we have focused on achieving high quality SMSN images in the second-range. The imaging speed can be further increased by lowering the targeted localization density or by allowing more than one active fluorophore in a diffraction-limited area and utilizing multi-emitter fitting algorithms for data analysis [10, 23–25].

The data presented here is limited to Alexa Fluor 647 in the thiol-based imaging buffer. While we believe that this combination accounts for the large majority of current SMSN applications, we want to point out that our conclusions cannot be directly transferred to other combinations of probes and buffer conditions since the photophysical properties can differ dramatically [14]. For example, it has been demonstrated that the photoswitching rates of oxazine dyes, such as ATTO dyes, can be controlled through the concentration of the reductant and the oxidant in the imaging buffer [26]. The quantum yield of oxazine dyes can also be improved using imaging buffer based on heavy water instead of regular water [27]. More generally, for dyes including Alexa Fluor 647, alternative imaging buffer compositions have been reported which improve, for example, the photon yield per emitter (i.e. the number of emitted photons before the molecule switches to a dark state)[28, 29]. Many other fluorescent probes and imaging conditions are designed for specific biological applications such as multi-color [14], live-cell [8, 10, 30–32] and high-throughput [33] SMSN. The protocols to optimize imaging parameters presented here can be easily adapted and applied to the characterization and optimization of SMSN under these conditions.

Furthermore, we want to emphasize that the optimal imaging conditions depend strongly on the sample structure, especially the labeling density. The higher the labeling density is, the lower the percentage of active fluorophores should be at any point in time to ensure that fluorescence emissions of individual molecules are spatially separated. An increase in the dimensionality of the sample (bulky vs. planar vs. fibrillar vs. sub-diffraction-sized clusters) will affect the local density of fluorophores non-linearly [34]. Thus, in bulky and densely labeled samples where surrounding fluorophores as well as fluorophores above/below the focal plane can lead to very large local probe densities, imaging conditions with lower percentages of active fluorophores are recommended. Our simulation of the fraction of fluorophores in the ON-state (**Fig 9C**) shows that, for Alexa Fluor 647 in the standard imaging buffer, lower percentages of active fluorophores can be achieved by increasing the excitation intensity.

Alternative illumination schemes such as HILO [35] or light-sheet illumination [36, 37] may be used to reduce the background introduced by fluorophores above/below the focal plane. However, the effect of the axially variable laser intensity on the percentage of active fluorophores in different intensity zones needs to be considered carefully since the total background level is proportional to the product of the average photon flux per fluorescence emission and the local density of active fluorophores. While the former decreases with decreasing laser intensity, the latter increases with decreasing laser intensity for our sample conditions (**Fig 9C**). However, for other conditions, for example when using photoactivatable fluorescent proteins, the density of active fluorophores may depend on laser intensity differently.

In this context, it is interesting to note that our simulations show that, upon irradiation with 642-nm light, the majority of the probe population transitions into the reversible dark states in ~ 1–2 s (**Fig 9A and 9B**), i.e. within a time frame that is much shorter than the course of a traditional SMSN imaging session. Any further reduction in the number of active fluorophores is attributed to photobleaching (or transition of fluorophores into dark states that are non-reversible over the course of imaging and behave effectively as a bleached state). Traditionally, many SMSN users include a ‘pre-bleaching’ step before the start of data recording, where the sample is illuminated with a low intensity (1–3 kW/cm^2^) of excitation laser light for about one minute till the density of active fluorophores is low enough for effective single-molecule localization[14]. Our simulations suggest that this ‘pre-bleaching’ step may indeed represent photobleaching of the sample rather than shelving of more molecules to the reversible dark states. Depending on the labeling density of the sample and the excitation intensity used, the amount of required photobleaching can be substantial. For example, in **Fig 9B**, the fraction of active fluorophores at equilibrium is about twice as high as the hypothetical threshold. In this particular case, half of the total probe population needs to be bleached before effective SMSN imaging can begin. Given the demand for maximum localization densities, this ‘pre-bleaching’ step is very unfavorable.

The work presented here leads to the conclusion that high intensities not only increase the speed of imaging, but also have the potential to lead to a more efficient use of the available probe molecules since fewer molecules need to be bleached before SMSN imaging can start. On the other hand, **Fig 5A** suggests that higher laser intensities may lead to a disproportionate rate of photobleaching which counteracts the benefit of high intensities described above. Thus, we recommend the following procedure for SMSN imaging to maximize the localization density in the final image: in the beginning of the experiment, high intensities (16–62 kW/cm^2^) should be applied to quickly push the density of active fluorophores below the threshold of efficient localization; after the threshold is reached, the excitation intensity could be gradually lowered to slowly increase the fraction of active fluorophores (**Fig 9C**) and thereby offset the loss of molecules caused by photobleaching. This procedure should not only yield the maximum localization density but also achieve it in the shortest possible time.

## Methods

### Instrumental setup

All experiments were performed on a custom-built SMSN instrument described previously [10]. Briefly, a 642-nm fiber laser (500 mW; MPB Communications) was first expanded and then focused into the back focal plane of a high-numerical-aperture (NA) oil-immersion objective (alpha Plan-Apochromat 100x, NA 1.46; Zeiss) to create Gaussian-distributed, wide-field illumination of the sample. Additionally, a 405-nm laser (CrystaLaser) was directed into the objective to illuminate the same area as the 642-nm light. Fluorescence emission was collected by the same objective, passed through a dichroic mirror (FF01-446/523/600/677; Semrock), a bandpass filter (ET 700/75; Chroma), and focused onto a sCMOS camera (Orca Flash 4.0; Hamamatsu Photonics). Relay lenses were used in the detection beam path so that the effective pixel size corresponding to the sample plane was 103 nm. The readout noise variance, gain and offset of the sCMOS camera were calibrated as described previously [10].

To stabilize the focus during data acquisition, a 940-nm fiber-coupled diode laser (LP940-SF30; Thorlabs) was introduced into the instrument in an objective-type total internal reflection beam path. Reflection from the coverglass-buffer interface was imaged onto a USB CMOS camera (DCC1545M; Thorlabs). The position of the reflected image was localized and used as feedback to adjust a piezo objective positioner (P721.CLQ; Physik Instrumente), keeping the axial focal drift to less than 10 nm.

### Laser intensity calibration

To calibrate the intensity of the 642-nm light at the sample, we used a red autofluorescence slide (Chroma) to determine the standard deviation (σ) of the Gaussian-distributed intensity profile (measured σ = 6.6 ± 0.3 μm, data not shown). Only the center of the illuminated area was used for data acquisition to ensure that the laser intensity varied less than 15% over the field of view (6.4 x 6.4 μm^2^). The reported laser intensities are the peak intensities (I_peak_) in the center of the field of view and were calculated from the power of the 642-nm light measured at the back aperture of the objective (P), the transmission rate (T) of the objective (T = 74%, provided by spec. sheet, confirmed by measurements) and the standard deviation of the Gaussian-distributed intensity profile (σ) using *I*_peak_ = *P*⋅*T*/2*πσ* ^2^ as derived below:

$$\begin{array}{c} I\left(x,y\right)=I_{\text{peak}}\cdot \;{\text{e}}^{-\frac{\left(x^{2}+y^{2}\right)}{2\sigma^{2}}}\\ P\cdot T=\int_{-\infty }^{+\infty }\int_{-\infty }^{+\infty }I\left(x,y\right)\text{d}\;x\;\text{d}\;y=I_{\text{peak}}\cdot 2\pi \sigma^{2}\\ I_{\text{peak}}=P\cdot T/2\pi \sigma^{2} \end{array}$$

We measured the laser intensity to fluctuate within ± 0.5% over 20 minutes.

### Sample preparation for Alexa Fluor 647-immunolabeled microtubules

COS-7 cells (ATCC) were grown in DMEM (phenol red-free, Life Technologies) with 10% FBS (Life Technologies) at 37°C with 5% CO_2_. Prior to imaging, cells were grown on 18 x 18 mm coverslips (170 ± 5 μm thickness). To label the microtubules, cells were washed three times with PBS and pre-extracted with 0.2% Saponin (Sigma) in Cytoskeleton Buffer (CSB, 10 mM MES pH 6.1 (Sigma), 150 mM NaCl, 0.5 mM MgCl_2_ (Sigma), 5 mM EGTA (Sigma), 5 mM glucose) for 1 min at room temperature. After aspirating the solution, the cells were fixed with 3% paraformaldehyde (PFA, Electron Microscopy Sciences) and 0.1% glutaraldehyde (Electron Microscopy Sciences) diluted in CSB for 15 min. Cells were washed three times for 3-min intervals with PBS and then permeabilized and blocked with blocking buffer (3% BSA from Sigma and 0.2% Triton X-100 in PBS) for 30 minutes while gently rocking. The buffer was aspirated and the cells were incubated with mouse monoclonal anti-α-tubulin antibody (Sigma T5168, 1:1000 dilution) in antibody buffer (1% BSA and 0.2% Triton X-100 in PBS) at room temperature for 1 h. Cells were washed three times for 3-min intervals using wash buffer (0.05% Triton X-100 in PBS) and incubated with Alexa Fluor 647 goat anti-mouse IgG (Life Technologies) in antibody buffer at 1:1000 dilution for 1 h. Cells were washed with the wash buffer for three 3-min intervals and post-fixed with 3% PFA and 0.1% glutaraldehyde diluted in CSB for 10 min. Samples were washed three times in PBS for 3-min intervals and stored in PBS at 4°C until imaging.

For imaging, the 18 x 18 mm coverslips were mounted cell-side down onto ~2 mm thick glass slides with a concave well (Cat. # 71878–05, Electron Microscopy Sciences) that held the imaging buffer and were sealed using dental glue (Picodent twinsil).

### Sample preparation for single-molecule experiments

Single-molecule experiments to characterize the photoswitching kinetics of Alexa Fluor 647 were performed using dye-labeled antibodies. Samples were prepared following previously published protocols [14]. Briefly, we suspended Alex Fluor 647 NHS ester (Life Technologies) in anhydrous dimethyl sulfoxide and mixed the dye with unlabeled goat anti-rabbit antibodies (Jackson ImmunoResearch) in 0.1 M aqueous sodium bicarbonate. Labeled antibodies were separated from free dye using Micro Bio-Spin P-30 chromatography columns (Bio-Rad Laboratories). We varied the concentration of Alexa Fluor 647 NHS ester in the labeling reaction to yield 0.13–0.3 dye molecules per antibody, which was measured using a UV-Vis spectrophotometer (Bio-Rad Laboratories). For imaging, the labeled antibodies were immobilized on coverslips (170 ± 5 μm thickness) at low densities (2–8 dye/μm^−2^). Coverslips were pre-cleaned by 15 minute sonication in 1 M potassium hydroxide, Mili-Q water, and 100% ethanol sequentially. The coverslips were mounted sample-side down onto glass slides in the same way as the microtubule samples.

### Imaging buffer for Alexa Fluor 647-labeled samples

Oxygen scavenging enzymes, catalase from bovine liver (Sigma) and glucose oxidase from Aspergillus niger (Sigma), were reconstituted in 20 mM Tris pH 7.4 (Sigma), 50 mM NaCl (Sigma) and 10 mM β-mercaptoethanol (β-ME, Sigma), and stored separately in 50% glycerol at -20°C at concentrations of 500 kU/mL catalase and 13.5 kU/mL glucose oxidase. The enzymes were diluted into 100 μL of imaging buffer (50 mM Tris pH 8.0, 50 mM NaCl (Sigma), 10% glucose, 143 mM β-ME) to 1 kU/mL catalase and 0.135 kU/ml glucose oxidase immediately before use.

### Single-molecule localization analysis

Fluorescence emission bursts from a single molecule may last for several camera frames and, if not corrected for, result in multiple localization events (**Figure D in S1 File**). This affects both localization precision and density through “over-counting.” To avoid this artifact, we used a three-step single-molecule analysis method. First, single emitters were identified from raw images and localized using the previously developed algorithms with sCMOS-specific noise model [10]. Next, localization events from neighboring frames were compared for their spatial and temporal distance. Localizations that were spatially separated by ≤ 25 nm (upper limit of localization precision) and temporally separated by ≤ 20 ms (temporal resolution from the lowest frame rate used, 50 fps) were assumed to stem from the same emission burst. Then, raw images corresponding to these localizations were cut out, added together and localized again. This time, the sCMOS noise model had to be adjusted according to the number of raw images that were added. For a single image combined from *n* raw camera frames, the variance of sCMOS readout noise for each pixel would be *n*-times as high as its characterized value in one frame. After rejecting localizations with uncertainties larger than 25 nm, a final list of localizations was generated, which was used to calculate the average localization precision, density of localized molecules, and to reconstruct SMSN images.

### Localization precision, number of photons per localization, background and FRC resolution

To report the average localization uncertainty or precision (**Fig 2A**), we estimated the uncertainty of each localization using a sCMOS-specific Cramér–Rao lower bound algorithm described previously [10], rejected localizations with uncertainties larger than 25 nm, and calculated the arithmetic mean. Data from four experiments repeated at the same imaging conditions were used to calculate the plotted mean values and the standard error of the mean (error bars).

The number of photons for each non-rejected localization event was averaged and reported as the average number of detected photons per localization (**Fig 2B**). Similarly, the number of background photons for each localization event was averaged and reported as the average number of background photons (**Fig 2C**). Background photons potentially include contributions from molecules out of focus, auto-fluorescence of the cell, cover slip or media and laser background.

The FRC values (**Fig 4A**) were calculated using software published by Nieuwenhuizen *et al*. with the FRC threshold set to 1/7 ≈ 0.143 as suggested in the paper [16].

### Localization density and the length of microtubules

To compare the localization densities of different samples (**Fig 3A and 3B**, **Fig 5A**, **Fig 7**and **Fig 8C**), we determined the number of localization events per 1 μm length of microtubule (MT) by dividing the total number of localizations (after rejecting localizations with uncertainties larger than 25 nm) by the length of the imaged MT. To extract the MT length from a super-resolution image, we first converted the reconstructed image to a binary image and extracted the outline of the binary image (**Figure E in S1 File**). We then calculated the perimeter of the MT outline by multiplying the number of pixels representing the outline and the effective pixel size of the reconstructed image. Finally, we estimated the MT length by dividing the perimeter of the MT outline by two, considering that the width of the MT outline (~ 100 nm) is much smaller than the length of each imaged MT (~ 3–10 μm) (See **S2 Software** for details).

### Derivation of the on/off-switching rates from single-molecule experiments

We imaged single-molecule samples at the same laser intensities as used for microtubule samples. At low intensities (1.0–16 kW/cm^2^), we recorded raw images at 200 fps to allow for both high temporal resolution and sufficient photon detection efficiency. At high intensities (31–97 kW/cm^2^), we recorded raw images at 800 fps because the ON-state lifetime is reduced to a few milliseconds and requires higher temporal resolution. For each intensity, ~1,600–6,000 events (from ~300–1,400 molecules) where a molecule switched from a fluorescent state to a reversible dark state were recorded in 20,000–24,000 raw frames. We calculated the ON-times by multiplying the number of frames with consecutive localizations with the camera exposure time. We calculated the OFF-times by multiplying the number of frames between two localizations of the same molecule with the exposure time.

From the photoswitching model (**Fig 6A**), we can derive that the distribution of ON-times follows an exponential function f_ON_(*t*) = *k*e^−*kt*^, where *k* is the switching rate from the singlet excited state to the triplet state, and that the distribution of OFF-times is a sum of three exponential functions, ${\text{f}}_{\text{OFF}}\left(t\right)=\sum_{i=1}^{3}a_{i}\lambda_{i}\;{\text{e}}^{-\lambda_{i}t}$, where *a*_*i*_ and *λ*_*i*_ are determined by the other switching rates (*k*_23_, *k*_34_, *k*_21_, *k*_31_ and *k*_41_ as in **Fig 6A**). From the distributions of on/OFF-times that we recorded in single-molecule experiments, we estimated *k*, *a*_*i*_ and *λ*_*i*_ by maximum-likelihood estimation, from which we further derived the switching rates at different intensities. The derivations are shown in detail below:

We denote P_ON_ (*t*) as the probability that a molecule is continuously in the ON-state from time 0 to time *t*. The probability for the molecule to switch off during a time interval ∆*t* is *k*_12_∆*t*. Thus, we can write,

$$\frac{{\text{dP}}_{\text{ON}}\;\left(t\right)}{\text{dt}}=-k_{12}\times {\text{P}}_{\text{ON}}\;\left(t\right)$$

The solution to the above equation with the initial condition P_ON_ (*t*) = 1 is,

$${\text{P}}_{\text{ON}}\left(t\right)={\text{e}}^{-k_{12}t}$$

Note that if a molecule stays on from time 0 to time *t*, its individual ON-state lifetime must be at least *t* [38]. We can therefore write,

$$\begin{array}{l} {\text{P}}_{\text{ON}}\left(t\right)\;=\text{Prob}\;(\text{ON-state lifetime}>\;t)\\ \text{Prob}\left(\text{ON-state lifetime}\leq t\right)=1-{\text{P}}_{\text{ON}}\left(t\right) \end{array}$$

We denote f_ON_ (*t*) as the probability density function of ON-state lifetime and we can write,

$${\text{f}}_{\text{ON}}\left(t\right)=\frac{\text{dProb}\;\left(\text{ON-state lifetim}\;\text{e}\leq \;t\right)}{\text{d}t}=-\frac{{\text{dP}}_{\text{ON}}\;\left(t\right)}{\text{d}t}=k_{12}\;{\text{e}}^{-k_{12}t}$$

Similarly, to derive the OFF-time distribution, we denote P_OFF_(*t*) as the probability that a molecule is in the OFF-state from time 0 to time *t*. We can write kinetic equations in terms of the probability of finding a molecule in a certain OFF-state at time *t* as,

$$\begin{array}{c} \frac{{\text{dP}}_{\text{T}}\;\left(t\right)}{\text{dt}}=-{\text{P}}_{\text{T}}\;\left(t\right)\times k_{21}-{\text{P}}_{\text{T}}\;\left(t\right)\times k_{23}\\ \frac{{\text{dP}}_{\text{D}}\;\left(t\right)}{\text{dt}}={\text{P}}_{\text{T}}\;\left(t\right)\times k_{23}-{\text{P}}_{\text{D}}\;\left(t\right)\times (k_{31}+k_{34})\\ \frac{{\text{dP}}_{\text{LLD}}\;\left(t\right)}{\text{dt}}={\text{P}}_{\text{D}}\;\left(t\right)\times k_{34}-{\text{P}}_{\text{LLD}}\;\left(t\right)\times k_{41} \end{array}$$

Using the Laplace transform, the solution to the above equations with the initial condition P_T_ (0) = 1, P_D_ (0) = 0, and P_LLD_ (0) = 0 is,

$$\begin{array}{c} {\text{P}}_{\text{T}}\;\left(t\right)={\text{e}}^{-\lambda_{1}t}\\ {\text{P}}_{\text{D}}\;\left(t\right)=\;\frac{k_{23}}{\lambda_{2}-\lambda_{1}}({\text{e}}^{-\lambda_{1}t}-{\text{e}}^{-\lambda_{2}t})\\ {\text{P}}_{\text{LLD}}\;\left(t\right)=\;k_{23}k_{34}\frac{1}{D}\left(A\;{\text{e}}^{-\lambda_{1}t}+B\;{\text{e}}^{-\lambda_{2}t}+C\;{\text{e}}^{-\lambda_{3}t}\right) \end{array}$$

Where

$$\begin{array}{c} \lambda_{1}=k_{21}+k_{23},\;\lambda_{2}=k_{31}+k_{34},\;\lambda_{3}=k_{41},\\ A=\lambda_{2}-\lambda_{3},\;B=\lambda_{3}-\lambda_{1},\;C=\lambda_{1}-\lambda_{2},\\ D=(\lambda_{2}-\lambda_{3})\lambda_{2}\lambda_{3}+(\lambda_{3}-\lambda_{1})\lambda_{1}\lambda_{3}+(\lambda_{1}-\lambda_{2})\lambda_{1}\lambda_{2} \end{array}$$

In analogy to the derivation of the probability density function of ON-state lifetime, if a molecule stays off from time 0 to time *t*, its individual OFF-state lifetime must be at least *t*.

Since,

$${\text{P}}_{\text{OFF}}\;\left(t\right)={\text{P}}_{\text{T}}\;\left(t\right)+{\text{P}}_{\text{D}}\;\left(t\right)+{\text{P}}_{\text{LLD}}\;\left(t\right)$$

The probability density function of the OFF-state lifetime is

$${\text{f}}_{\text{OFF}}\;\left(t\right)=\;\frac{\text{dProb}\;\left(\text{OFF-state lifetim}\;\text{e}\leq \;t\right)}{\text{d}t}=-\frac{{\text{dP}}_{\text{OFF}}\;\left(t\right)}{\text{d}t}=-\frac{\text{d}[{\text{P}}_{\text{T}}\;\left(t\right)+{\text{P}}_{\text{D}}\;\left(t\right)+{\text{P}}_{\text{LLD}}\;\left(t\right)]}{\text{d}t}$$

Substitute P_T_(*t*), P_D_(*t*) and P_LLD_(*t*) with their respective solutions, we can write,

$$\begin{array}{l} {\text{f}}_{\text{OFF}}\;\left(t\right)=a_{1}\lambda_{1}\;{\text{e}}^{-\lambda_{1}t}+a_{2}\lambda_{2}\;{\text{e}}^{-\lambda_{2}t}+a_{3}\lambda_{3}\;{\text{e}}^{-\lambda_{3}t}=\sum_{i=1}^{3}a_{i}\lambda_{i}\;{\text{e}}^{-\lambda_{i}t}\;,\;a_{1}+a_{2}+a_{3}=1\\ a_{1}=1+\frac{k_{23}}{\lambda_{2}-\lambda_{1}}+\frac{k_{23}k_{23}A}{D}\;,\;a_{2}=\;-\frac{k_{23}}{\lambda_{2}-\lambda_{1}}+\frac{k_{23}k_{23}B}{D}\;,\;a_{3}=\frac{k_{23}k_{23}C}{D} \end{array}$$

### Artificial microtubules and artificial structures with low labeling density

The artificial microtubules (shown in **Fig 8A**, used for plotting **Fig 7**and **Fig 8C**), consisted of two lines of molecules. The distance between adjacent molecules in the same line was set to 5 nm and the distance between the two lines to 40 nm. In the case of the artificial grid structure with sparse labeling (shown in **Fig 8B**, used for plotting **Fig 8C**), the distance between adjacent molecules was set to 800 nm.

### Simulation of SMSN imaging of artificial structures

To simulate raw camera frames of SMSN imaging of artificial structures, we first simulated a temporal trace of the photophysical states for each fluorophore over the complete time course. We then segmented these traces into camera frames according to the exposure time and plotted the images of emitting molecules using a pixel-integrated 2D Gaussian model. The number of photons detected in each camera frame depends on both the photon flux, which was determined based on experimental results by dividing the number of detected photons per switching cycle by the ON-state lifetime, and how long the molecule stays on within the frame. The number of background photons depends on both the camera exposure time and the laser intensity. Images of the simulated structure were first generated with Poisson noise; then pixel-dependent sCMOS noise was added to each pixel using the noise parameters characterized from a physical subregion of the actual sCMOS camera used (See **S1 Software**). These simulated data were then analyzed with the single-molecule analysis method described above.

### Reconstruction of super-resolution images

In all SMSN images shown, localization estimates were binned into two-dimensional (2D) histogram images with 5.2 nm x 5.2 nm pixel size. To aid visualization, each resulting image was convolved with a 2D Gaussian kernel with σ = 1.5 pixels. Sample drift during data acquisition in the x, y directions was corrected through correlation of the images reconstructed from every 2,000 frames.

## Supporting Information

S1 File This file contains supplementary figures. Example distribution of OFF-times with fit (**Figure A**). OFF-time distributions at different excitation intensities (**Figure B**). Example experimental data showing rejection of overlapping signals at low frame rates (**Figure C**). Examples of fluorescence emission events that lasted for several camera frames and were repeatedly localized (**Figure D**). Estimation of the length of imaged microtubules (**Figure E**).
(DOCX)

S1 Movie Representative example of SMSN dataset acquired at conventional imaging conditions (50 fps, 3.9 kW/cm^2^) corresponding to the reconstructed image shown in Fig 4B. Scale bar: 1 μm.
(AVI)

S2 Movie Representative example of SMSN dataset acquired at high speed conditions (800 fps, 62 kW/cm^2^) corresponding to the reconstructed image shown in Fig 4C. Scale bar: 1 μm.
(AVI)

S1 Software Simulation of SMSN image frames. A Matlab software package for simulating raw sCMOS or EMCCD camera frames of SMSN imaging of artificial microtubules.
(ZIP)

S2 Software Extraction of microtubule length. A Matlab software package for automated calculation of microtubule length based on the reconstructed images.
(ZIP)
