# Supplementary material for: Quantifying and Optimizing Single-Molecule Switching Nanoscopy at High Speeds
Source: PLoS One. 2015 May 26;10(5):e0128135. doi: 10.1371/journal.pone.0128135 (PMC4444241; doi:10.1371/journal.pone.0128135)
Supplement: S1 File — Example distribution of OFF-times with fit (Figure A). OFF-time distributions at different excitation intensities (Figure B). Example experimental data showing rejection of overlapping signals at low frame rates (Figure C). Examples of fluorescence emission events that lasted for several camera frames and were repeatedly localized (Figure D). Estimation of the length of imaged microtubules (Figure E). (DOCX) [file pone.0128135.s001.docx]

**Supplementary Figures**

**
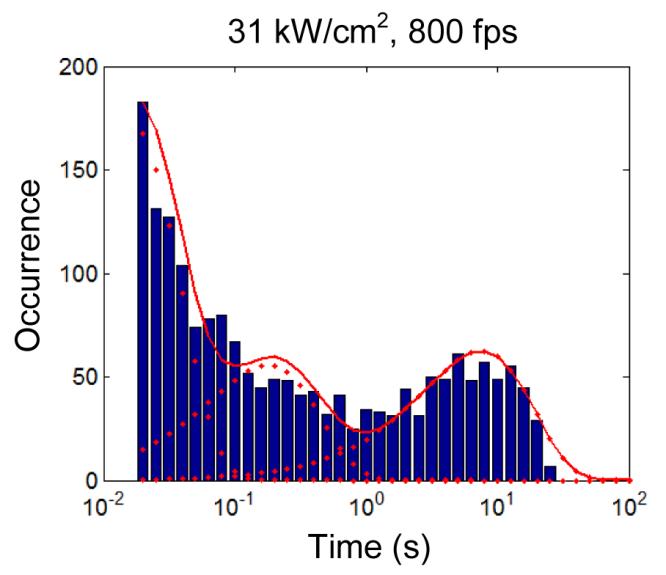
**

**Figure A.** Example distribution of OFF-times with fit. The OFF-times we observed in single-molecule experiments cover a wide range from several milliseconds to tens of seconds. As suggested by Sigworth and Sine et al.[[1](#_ENREF_1)], plotting the distribution of the logarithm of the OFF-times rather than the distribution of OFF-times makes the data and its fit easier to interpret. The distribution of OFF-times can be fit with a mixture of three exponential distributions (blue bar: data, red solid line: fit function, red dot line: each individual exponential component of the fit function, see **Methods** for derivation of the fit function).

[1] Sigworth FJ, Sine SM (1987) Data transformations for improved display and fitting of single-channel dwell time histograms. Biophys J 52: 1047-1054.

**
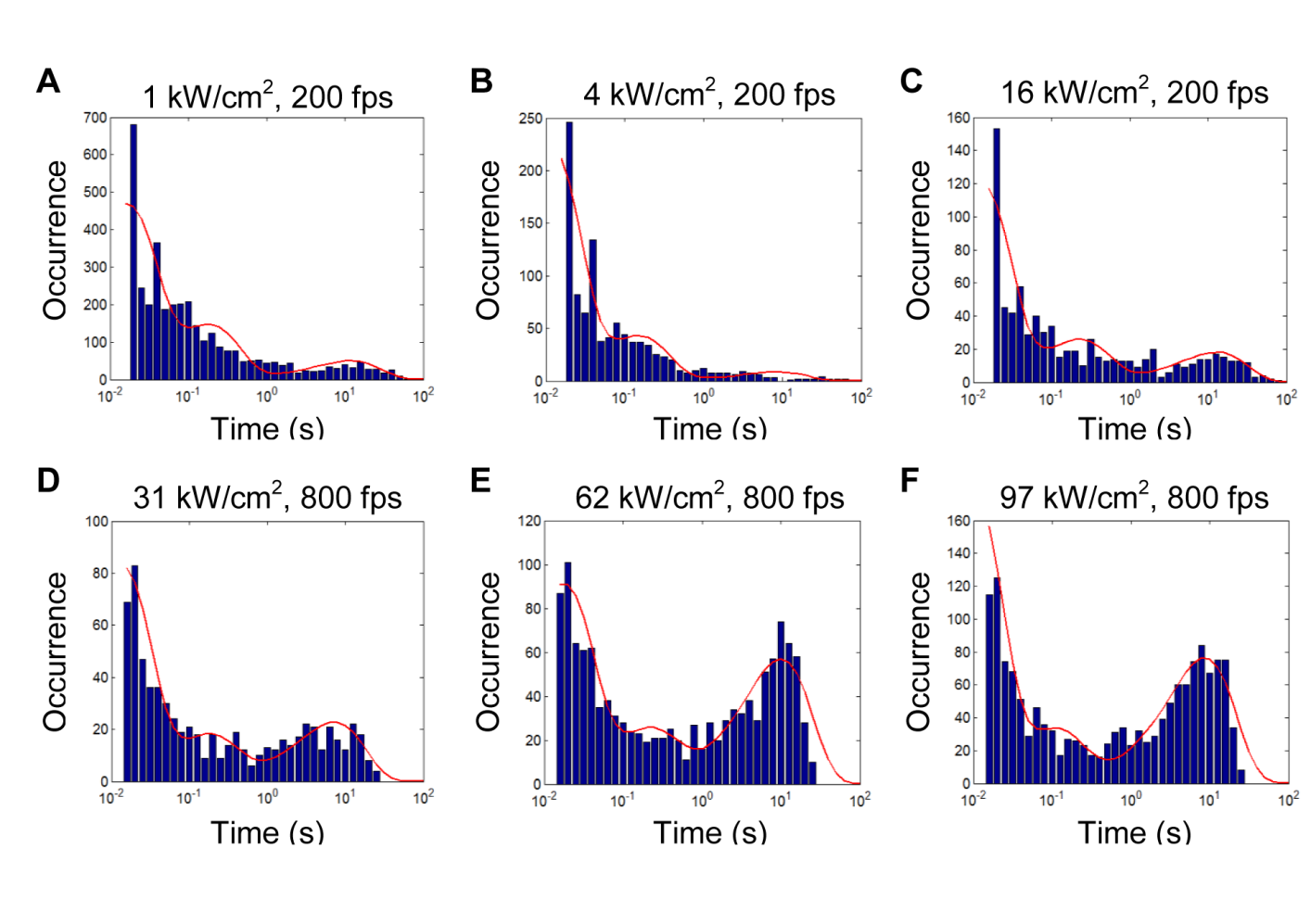
**

**Figure B.** OFF-time distributions at different excitation intensities from 1 – 97 kW/cm^2^.


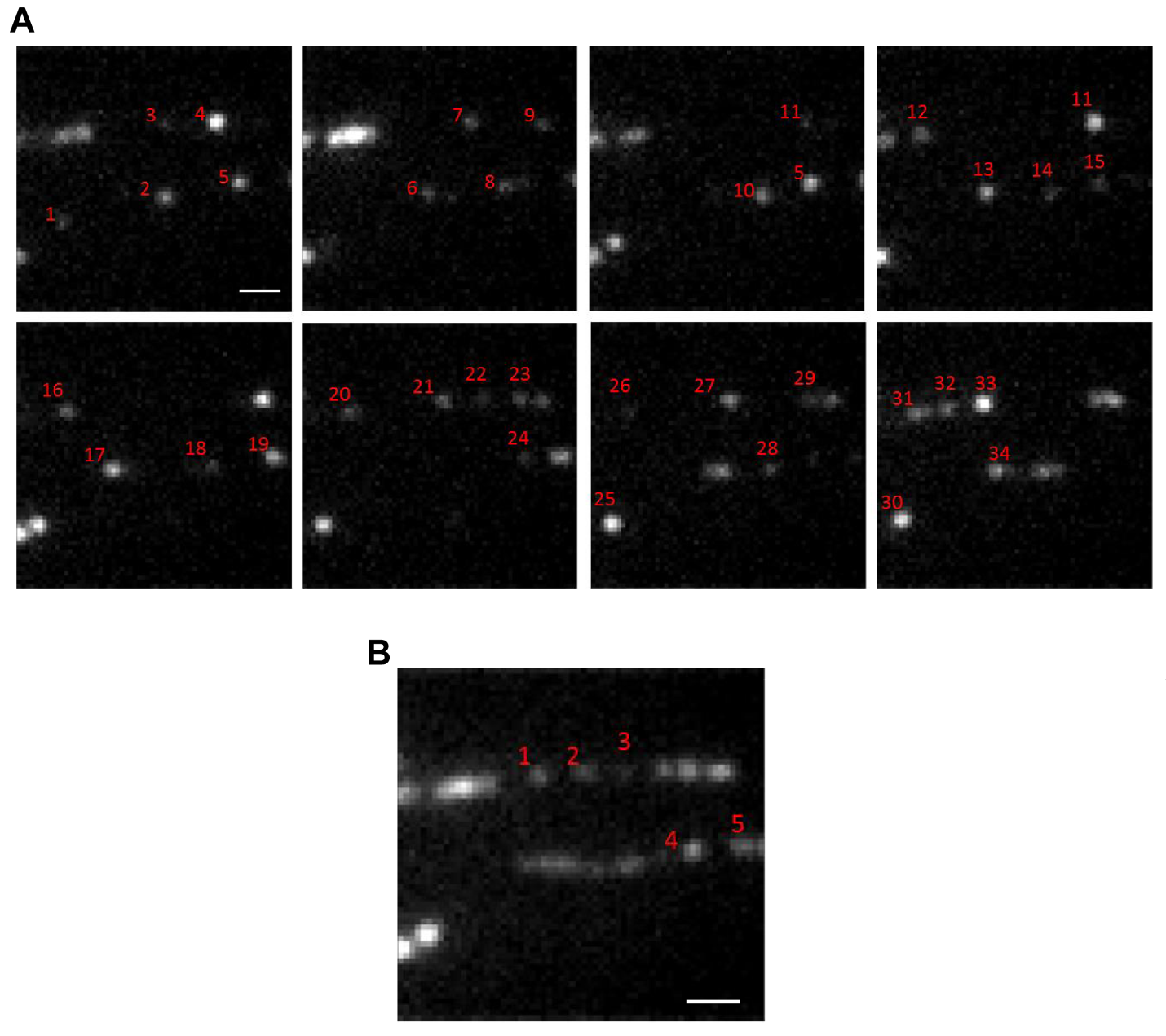


**Figure C.** Example experimental data showing rejection of overlapping signals at low frame rates. (**A**) Eight frames of SMSN raw images were acquired at 800 fps with 97 kW/cm^2^ of 642-nm light over 10 ms. 34 molecules were identified as single molecules and localized (red numbers). (**B**) An artificial single frame image generated by adding frames 1-8 from (**a**) to simulate recording of the same signal at 100 fps. Only 5 molecules were identified as single molecules because of overlapping of signals from neighboring molecules. Scale bars: 1 µm.


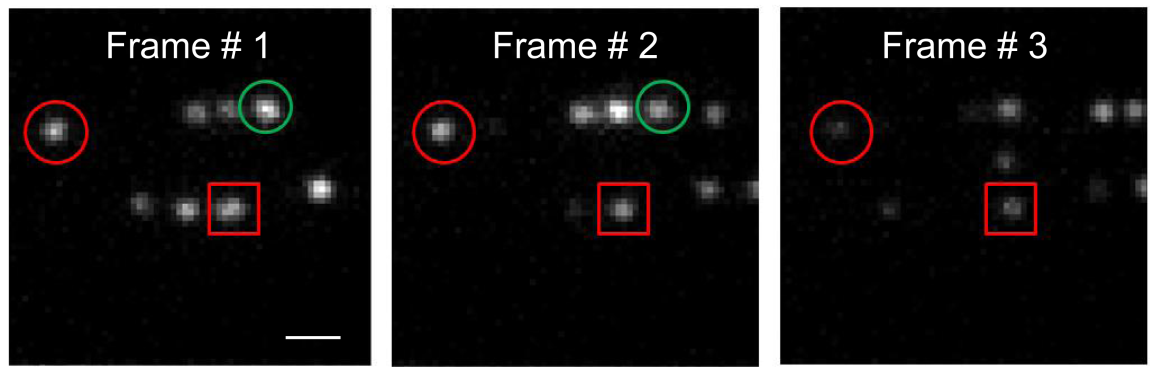


**Figure D.** Examples of fluorescence emission events that lasted for several camera frames and were repeatedly localized. As illustrated, emission events were localized two (green circle) or three times (red circle and red box) over consecutive frames. This phenomenon would affect both localization precision and density if not corrected for. To avoid this artifact, we grouped photons from the same emission event in our single-molecule analysis (see **Methods** for details). Scale bar: 1 µm.


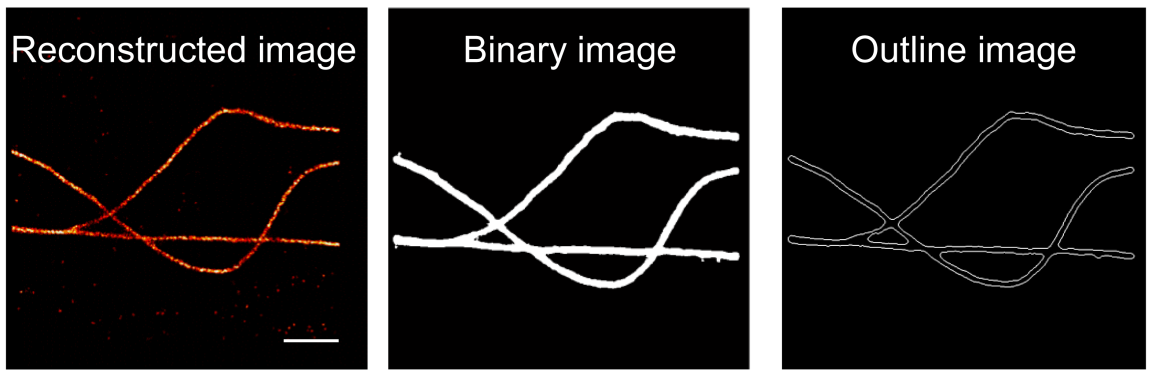


**Figure E.** Estimation of the length of imaged microtubules. The length of a microtubule was determined from the outline of the binary conversion of the reconstructed image (see **Methods** for details). Scale bar: 1 µm.
